# Fabrication of Hard–Soft Microfluidic Devices Using Hybrid 3D Printing

**DOI:** 10.3390/mi11060567

**Published:** 2020-06-01

**Authors:** Carlos Ruiz, Karteek Kadimisetty, Kun Yin, Michael G. Mauk, Hui Zhao, Changchun Liu

**Affiliations:** 1 Department of Mechanical Engineering and Applied Mechanics, University of Pennsylvania, 220 South 33rd St. Philadelphia, PA 19104-6315, USA; nikobaul@gmail.com (C.R.); karteek.kadimisetty@gmail.com (K.K.); kyin@uchc.edu (K.Y.); mmauk@seas.upenn.edu (M.G.M.); 2 Department of Biomedical Engineering, University of Connecticut Health Center, 263 Farmington Avenue, Farmington, CT 06030, USA; 3 Department of Mechanical Engineering, University of Nevada, Las Vegas, NV 89154, USA; hui.zhao@unlv.edu

**Keywords:** 3D-printing, microfluidics, prototyping, point-of-care diagnostics, nucleic acid amplification test

## Abstract

Widely accessible, inexpensive, easy-to-use consumer 3D printers, such as desktop stereolithography (SLA) and fused-deposition modeling (FDM) systems are increasingly employed in prototyping and customizing miniaturized fluidic systems for diagnostics and research. However, these 3D printers are generally limited to printing parts made of only one material type, which limits the functionality of the microfluidic devices without additional assembly and bonding steps. Moreover, mating of different materials requires good sealing in such microfluidic devices. Here, we report methods to print hybrid structures comprising a hard, rigid component (clear polymethacrylate polymer) printed by a low-cost SLA printer, and where the first printed part is accurately mated and adhered to a second, soft, flexible component (thermoplastic polyurethane elastomer) printed by an FDM printer. The prescribed mounting and alignment of the first-printed SLA-printed hard component, and its pre-treatment and heating during the second FDM step, can produce leak-free bonds at material interfaces. To demonstrate the utility of such hybrid 3D-printing, we prototype and test three components: i) finger-actuated pump, ii) quick-connect fluid coupler, and iii) nucleic acid amplification test device with screw-type twist sealing for sample introduction.

## 1. Introduction

Since the advent of the allied fields of lab-on-a-chip (LOC), microfluidics, and point-of-care (POC) diagnostics close to 30 years ago, there has been a considerable worldwide effort to develop microfluidic-based devices for medical diagnostics tests and other portable, miniaturized assays for use outside of laboratories [1,2,3,4,5,6,7]. Yet, there is still no broad consensus on the best materials, designs, and fabrication methods for many of these technologies. Microfluidic devices have been made out of silicon, glass, soft and hard polymers, and more recently, paper or other porous materials, using fabrication techniques including microfabrication processes adapted from semiconductor technology (e.g., lithography), computer numerical control (CNC) milling, hot embossing, laser cutting, and injection molding [8,9,10,11]. More recently, 3D printing has been utilized for making microfluidic and other LOC devices. 3D printing is an additive manufacturing technology where parts are built up, layer by layer, through selective addition or transformation of feedstock material in a computer-controlled optical or electromechanical desktop system. 3D printing can accelerate product development and enhance the productivity of research and engineering in many technology areas, including biomedical devices and applied chemistry.

Three-dimensional printing has important advantages for the design and development of microsystems and microfluidic devices. 3D printing is very suitable for customizing devices for different applications, small-lot production runs for clinical and field trials where diagnostic performance and reliability can be assessed, getting quick feedback from clinicians and other healthcare workers, as well as patients using companion diagnostic devices to monitor their treatment. In principle, with 3D printing, microfluidic device engineers can use computer-aided design (CAD) software to specify part geometry, and print working prototypes suitable for testing within a ~24-h cycle. For microfluidics applications, it is often desirable for the materials to be transparent to facilitate on-chip optical measurements, including UV and short optical wavelengths (350–500 nm) as needed for fluorescence detection, which further requires low background (auto) fluorescence. 3D-printed microfluidic devices must be compatible with operating temperatures that may be as high as 100 °C for chips incorporating polymerase chain reaction (PCR), or 65 °C for chips supporting isothermal nucleic acid amplification [12].

To increase the functionality of the microfluidic devices, different 3D printing methods have been used to prototype multi-material microfluidic devices. For example, Anderson et al. described 3D printing methods that could accommodate insertion of a membrane and off-the-shelf connector fittings in hard-plastic fluidic devices post-printing [13]. Alapan et al. developed hybrid microfluidic devices utilizing 3D printing and laser micromachined lamination to make integrated manifolds to reduce the need for assembly [14]. Gaal et al. reported fabrication of 3D-printed microfluidic devices by interrupting the fused-deposition modeling (FDM) and manually inserting different materials such as paper, wire, and glass [15]. Duong and Chen used solvent bonding to attach transparent acrylic components to FDM-printed Acrylonitrile Butadiene Styrene (ABS) for hybrid microfluidic devices [16]. Li et al. reviewed increased functionalities of multi-material micro devices by “print-pause-print methods” to insert external elements into 3D-printed structures [17]. In some microfluidic applications, both hard and soft materials are required to build various hard–soft hybrid, microfluidic devices, such as microvalves [18], microfluidic-based biosensor flow cell [19] and cell culture microfluidic device [20]. However, typically, a consumer 3D printer prints only one material at a time, and the production of hybrid devices integrating both rigid (‘hard’) and flexible (‘soft’) materials is challenging, although more versatile 3D printers are available to specialists for microfluidic device fabrication [21,22,23,24]. While more sophisticated 3D printers are entering the market or under development, these are, of course, more expensive and thus are not readily accessible to occasional users and non-specialists.

Here, we described a two-stage 3D printing process to produce hybrid microfluidic devices incorporating both hard and soft materials using inexpensive 3D consumer printers. A printed hard component is first made by a stereolithography (SLA) printer, and then transferred to a second FDM printer, where the soft printed component is appended by the FDM printing. As a demonstration of capabilities, we designed, fabricated and tested three different types of hybrid microfluidic devices: (i) a finger-actuated pump, (ii) a microfluidic quick connect component, and (iii) a microfluidic reactor chip with screw-seal sample inlet ports, which is used to host isothermal amplification and detection of *Salmonella typhimurium* (*S. Thyphi*) DNA by loop-mediated isothermal amplification (LAMP) [25]. 

## 2. Materials and Methods

### 2.1. Chemicals and Materials

Poly (ethylene glycol) 8000 (PEG) was obtained from Sigma-Aldrich (St. Louis, MO, USA), RT-PCR grade water from Ambion, Inc. (Austin, TX, USA), intercalating EvaGreen^®^ fluorescent dye from Biotium, Inc. (Hayward, CA, USA), Isothermal Master Mix (ISO- 001nd) used in LAMP reaction buffer from OptiGene (Horsham, UK), WarmStart^®^ colorimetric LAMP 2 X master mix from New England Biolabs (Ipswich, MA, USA). Thermoplastic polyurethane(TPU) filament, Flex Seal and Rust-Oleum Clear Gloss Enamel spray were purchased from Amazon. The LAMP assay used here, including primer sequences, is described in more detail in previous literature [25].

### 2.2. Fabrication of Hard–Soft Microfluidic Devices Using Hybrid 3D Printing

To fabricate our hard–soft microfluidic devices, we first used an inexpensive, high-definition Stereolithographic (SLA) laser-based Form 2, 3D printer (Formlabs, Inc., Somerville, MA, USA) to print the hard/rigid parts of the hybrid microfluidic devices. Clear methacrylate based resin (Forms labs, FLGPCL02) was used for prototyping. A dimensioned Computer-Aided Design (CAD) of the part was first created using Autodesk 123D or SolidWorks™ software, and converted to STL format. The STL files were uploaded to the Form2 unit and printed with 50-μm resolution. Then, printed parts were removed from the build platform of the printer and immersed in 99% isopropanol (IPA) for 10 min followed by 5-min ultra-sonication to remove any uncured resin. The printed parts were then thoroughly washed with water, and air dried. To improve the transparency of the SLA-printed parts for optical detection, the surfaces of the printed parts were coated with heat-resistant acrylic spray (Rust-OleumClear Gloss™ Enamel spray), which produced optically transparent surfaces.

To fabricate the soft/flexible parts, the hard SLA-printed parts of the microfluidic devices were mounted with a high-temperature adhesive to attach their bases to a glass plate. This facilitates the mated printing of the soft/flexible TPU material by the FDM 3D printing with a Monoprice Maker Ultimate™ 3D Printer. The FDM printer has 200 mm × 150 mm × 150 mm buildspace and 0.45-mm nozzle diameter (working temperature: 250 °C). Its XY accuracy is 0.01 mm, and Z accuracy 0.0025 mm. Prior to the initiating the extrusion printing of the soft material, the plate was heated to 60 °C for 30 min in order to preheat the hard parts, which improves the adhesion at the interface of the hard/soft material. A Z-axis offset with respect to the height of the component was applied: a 1.0-mm Z-offset compensates for the thickness of the glass plate, and an additional 0.5mm Z-offset compensates for thickness of the adhesive and any variations in mounting height. The FDM printer created an initial 1-layered outline with a 1-mm offset from the perimeter of the device prior to device placement on the substrate. This was used for initial alignment as the programmed outline is assumed to be more accurate after homing than a physical measurement, which could introduce human error.

**Figure 1:**
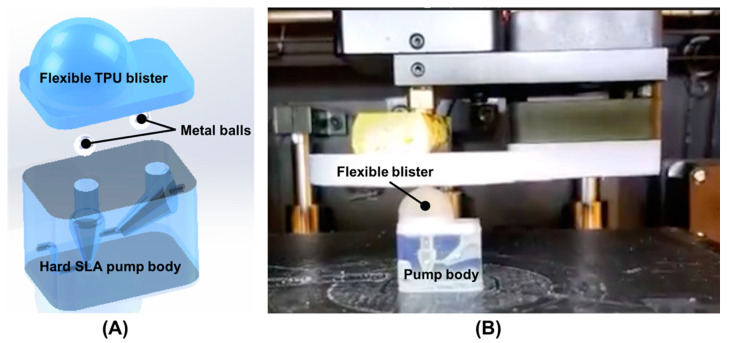
(**A**) Schematic illustration of the 3D-printed, hard–soft micropump that was powered by a finger. It consists of one hard SLA-printed pump body, two metal balls and one flexible TPU blister. (**B**) 3D printing of the flexible TPU blister on the hard SLA-printed pump body which was pre-filled with two metal balls before FDM 3D printing.

**Figure 2:**
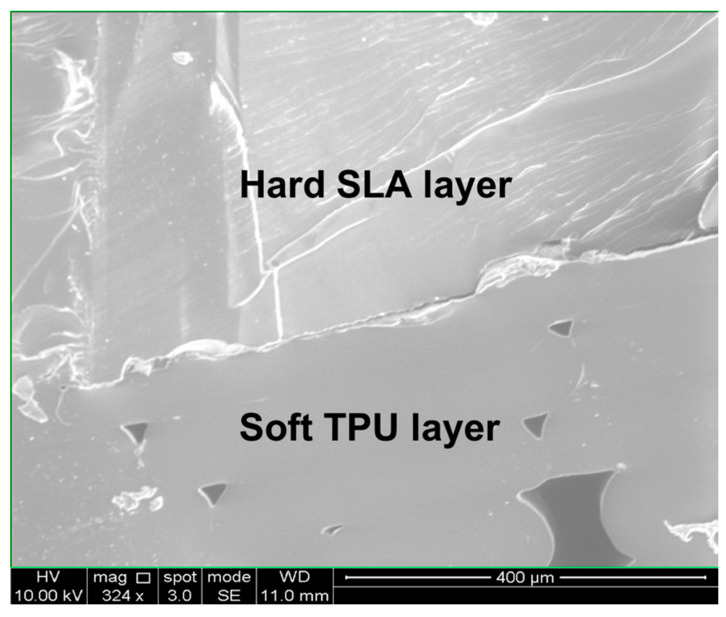
SEM image of the cross-section of the interface between the hard SLA-printed layer and soft TPU layer.

Figure 1A is a schematic illustration of our finger-powered, hard–soft, 3D printed micropump comprising a hard SLA-printed pump body, two metal balls, and one flexible TPU blister. The flexible TPU blister part was FDM printed on the hard SLA-printed pump body after inserting two metal balls (Figure 1B). Then, the surface of the TPU layer was coated with a clear coat of Flex Seal™. The cross-section interface between the hard SLA-printed layer and soft TPU layer was viewed using a Scanning Electron Microscope (SEM) equipped with a focused ion beam (FIB) (FEI Strata DB325) as shown in Figure 2. The voids result from the layer-by-layer build of the part, yet the first made a complete seal and fused sufficiently. Flex Seal was used to mitigate risk of TPU failure due to excessive compression. 

In the 3D-printed, microfluidic LAMP chip, the LAMP reactor volumes were about 25 μL each. To improve the biocompatibility of the LAMP reactors, the microfluidic reactors were passivated with a coating by filling and soaking the microfluidic reactors for 1 h with 2% aqueous Poly Ethylene Glycol (PEG 8000), followed by water rinsing and heated drying at 60 °C for 1 h. This treatment prevents non-specific adsorption of LAMP reaction components, and thus helps maintain good amplification efficiencies [12]. The PEG-coated devices were then stored at room temperature for later use.

### 2.3. Hybrid 3D-Printed LAMP Reactor Assay Protocol

In our hybrid LAMP reactor chips, we used a combined fluorescence-colorimetry detection for real-time and end-point monitoring of the LAMP reaction. We tested the LAMP chips with *Salmonella typhimurium (S. Thyphi)* genomic DNA (gDNA) and previously reported LAMP primers [25]. This organism is an important pathogen in food safety, which could represent a typical application of this device. The LAMP reaction mixture includes: 12.5 µL WarmStart^®^ colorimetric LAMP master mix, 1 µL Eva-Green^®^ fluorescent DNA-intercalating dye, 1.3 µL primer mix (the final concentrations of F3/B3, FIP/BIP and LF/LB were 0.2, 1.6, and 0.4 μM, respectively), 9.2 µL of PCR-grade water and 1 µL of sample. For positive samples, water spiked gDNA at concentrations corresponding to 5000 CFU (colony forming units) /reaction down to 100 CFU/reaction were tested. Pure water sample was used as negative control. Samples were introduced through a 100 µl pipette tip, and the reactor was sealed by means of the 3D-printed hard–soft screw cap.

After the microfluidic reactor chip was filled with samples, the device was placed on a VWR^®^ mini block heater to perform isothermal amplification at 64 °C (± 1 °C) for 1 h. A portable USB fluorescence microscope (AM4113T-GFBW Dino-Lite Premier, AnMo Electronics, Taipei, Taiwan) mounted through a viewing port of a 3D-printed dark box positioned above the chip to monitor fluorescence. Images of the reactor fluorescence were captured at one-minute intervals over a 1-h reaction and recorded with the DinoCapture^TM^ software. The fluorescence image intensities were analyzed by using MatLab^®^ software to yield normalized average fluorescence intensities from each reactor area. Normalized fluorescent intensities were plotted against time to obtain real-time amplification curves. Real-time monitoring with the DinoLite produces very similar results compared to LAMP performed on a benchtop PCR instrument [12]. Colorimetric detection was recorded at the end of LAMP incubation when the 3D-printed microfluidic chip was taken outside of the heater.

## 3. Results and Discussion

### 3.1. Finger-Powered, Hard–Soft 3D-Printed Micropump

**Figure 3:**
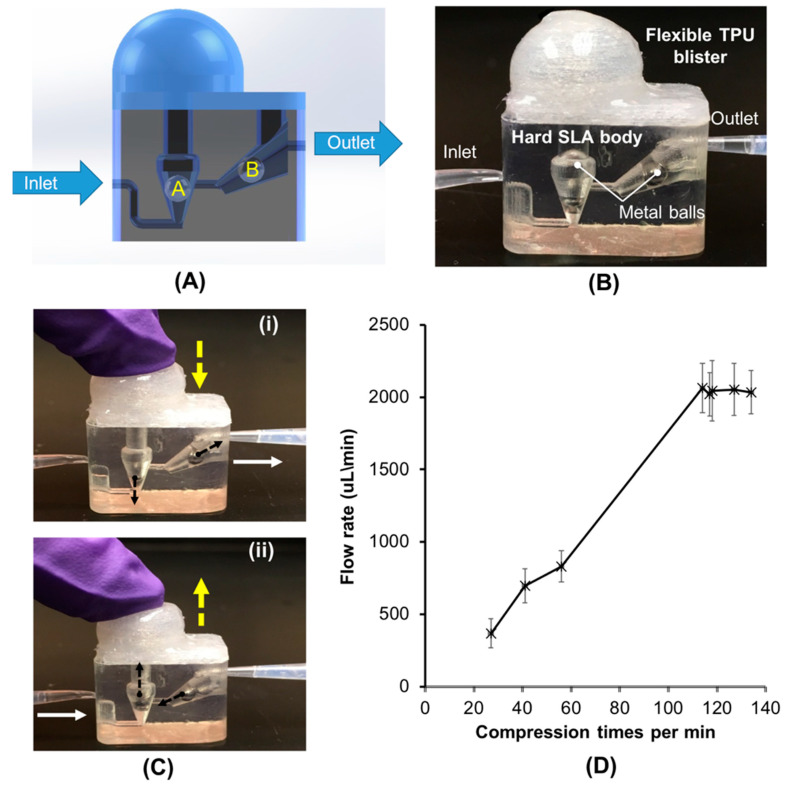
Finger-powered, hard–soft hybrid micropump. (**A**) Schematic illustration of the work principle of the hybrid micropump. (**B**) A photograph of the hard–soft hybrid pump. (**C**) A sequence of images illustrating the finger-powered pumping process in the hybrid micropump. The water containing food dye was used in this test. (**D**) The flow rate (µL/min), as a function of function of the compression times (expressed in terms of times per min). n = 3.

Figure 3A shows the working principle of our finger-actuated, hard–soft 3D-printed micropump. The pump operates by propagating fluid through two integrated check valves. To create the check valves, one metal ball with a 3-mm diameter was placed in each inlet and outlet channel. Figure 3B is a photograph of the hard–soft 3D-printed micropump with a volume of ~780 µL. The inlet valve is vertically oriented from the base and coincident to the axis of the inlet. The outlet valve’s channel had a 30 ° pitch to compensate for the mass of the ball. After the hard pump body was printed, two metal balls are inserted in their respective locations. Metal balls were used instead of glass or plastic spheres due to their higher mass and better spherical uniformity, both of which prevent backflow. 

Figure 3C shows a sequence of images illustrating the finger-powered pumping process in the hybrid micropump. To activate the micropump, the micropump is primed (pre-filled) with water. When the blister is compressed by a finger (Figure 3C-i), the inlet ball A stays seated to prevent backflow into the inlet channel, while outlet ball B rises to a position in the flared channel, permitting flow around, to the outlet. The blister is then released, creating a partial vacuum, and the inlet ball A rises, allowing new fluid to enter the blister and pump chamber. Meanwhile, the ball B is seated to block the outlet channel and to prevent black flow into the pump body (Figure 3C-ii). The net result is that in one cycle of compressing and releasing the flexible blister, a charge of liquid is propelled from the inlet into the outlet. We evaluated and determined the performance of our finger-powered, hard–soft hybrid micropump. Figure 3D, shows the averaged flow rate increases with cycle rate, and that the flow rate plateaus when the compression frequency is above approximately 100 cycles per min. With our hard–soft hybrid micropump, we can achieve a maximum flow rate greater than 2000 µL /min, which is suitable for most microfluidic-based biomedical applications. 

### 3.2. Hard–Soft Hybrid Microfluidic Connector

**Figure 4:**
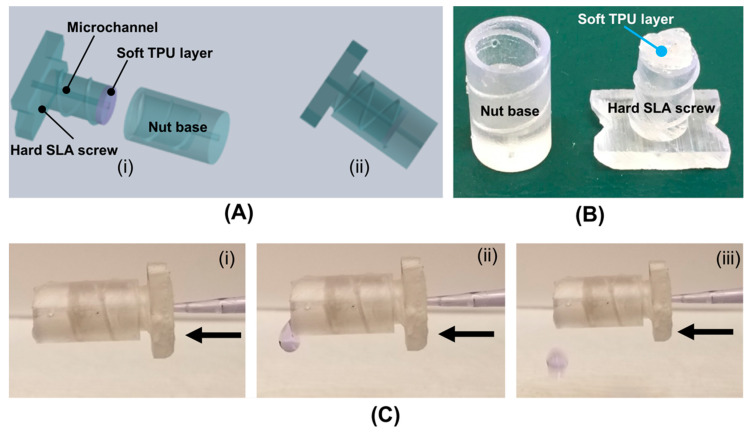
(**A**) Schematic illustration of the hard–soft hybrid microfluidic connector. (**B**) A photo of the 3D-printed nut case and hard–soft screw with a microchannel. (**C**) A sequence of images illustrating the fluid flowing process in the hard–soft hybrid connector.

To demonstrate the microfluidic quick connection application, we designed, fabricated and tested a hard–soft hybrid microfluidic connector (Figure 4A,B) made by 3D printing. The 3D-printed connector consists of two parts: (i) one hard SLA-printed nut case with a fluid outlet, and (ii) one hard–soft hybrid threaded screw with a soft TPU layer, which serves as a sealing material. To transport liquid, the hybrid screw contains a microfluidic channel with a diameter of 0.9 mm. The connector was tested by forcing water to flow through it. To better observe flow and any possible leakage, food dye was added to the water. Figure 4C shows a sequence of images illustrating the fluid flowing through the microfluidic connector. As shown in Figure 4C, no liquid leakage was observed in our hard–soft hybrid microfluidic connector, and the ready-to-use, as-printed hard–soft hybrid microfluidic connector is suitable for simple and rapid microfluidic connection, eliminating need for post-printing assembly or additional hardware.

### 3.3. Molecular Detection on 3D-Printed Microfluidic Reactor Chip with the Hard–Soft Threaded Screw Sealer

**Figure 5:**
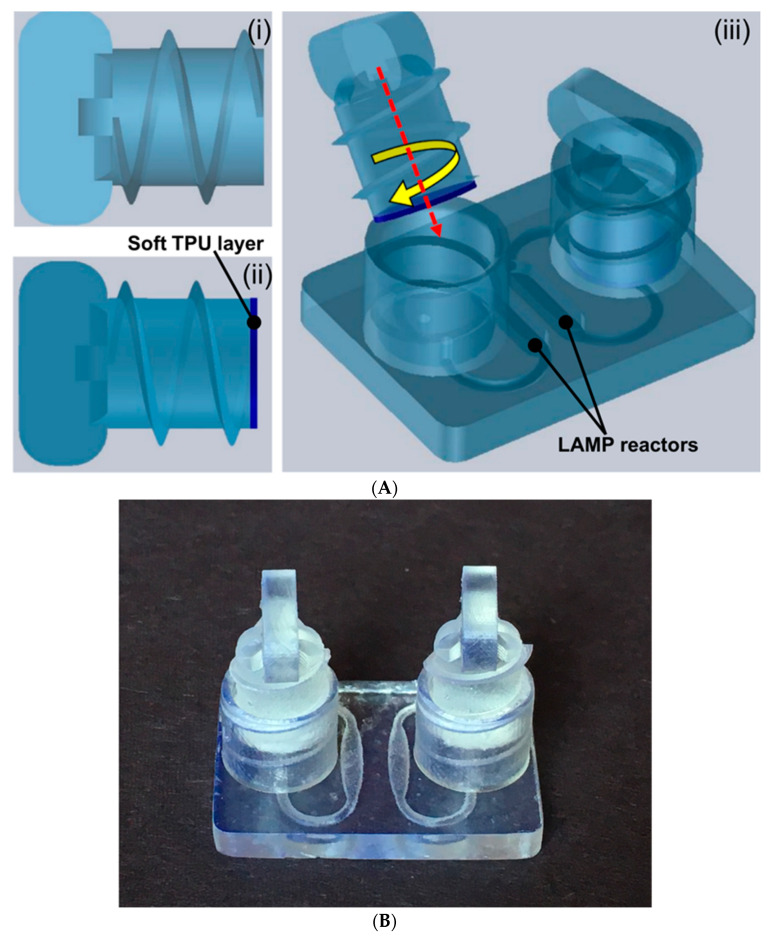
(**A**) Schematic illustration of the 3D-printed, hard–soft microfluidic LAMP chip platform for molecular detection. (i) Hard SLA-printed threaded screw sealer. (ii) Hard–soft threaded screw sealer with soft TPU material. (iii) Assembled hard–soft microfluidic LAMP chip platform. (**B**) Photo of the 3D-printed microfluidic LAMP chip platform with two hard–soft threaded screw sealers.

Nucleic acid (DNA, RNA) amplification tests that are capable of rapidly identifying the presence of pathogens with high selectivity and sensitivity are important enablers for enhanced infectious diseases management and accessible healthcare [26,27,28,29,30]. Such platforms that can be operated at the point-of-care with less complexity are needed to facilitate early treatment options and improve patient outcomes. Miniaturized microfluidic platforms facilitate adaptation, implementation and integration of multiple complex operations into a compact package for simple operation by non-experts, and wider use in resource limited settings. While most of the current microfluidic platforms for nucleic acid detection have successfully streamlined complex operations [31,32], they still require leakage-free sealing of the inlet and outlet to prevent liquid evaporation during the controlled heating process for enzymatic amplification, such as LAMP. As a biomedical application demonstration of hard/soft hybrid printing, we provide a proof of concept for a 3D-printed hybrid microfluidic device for a LAMP-based nucleic acid amplification test of *Salmonella typhimurium* (*S. Thyphi*) gDNA. The 3D-printed hard–soft threaded screw affords fast, reliable, leak-free sealing of LAMP amplification reactors without significant additional complexity and can be readily adapted to multiple LAMP reactors (multiplexing) or related applications. The 3D-printed microfluidic chip platform comprises: (i) two hard–soft 3D-printed screw sealers, and (ii) one 3D-printed LAMP reactor chip (Figure 5A). The microfluidic reactor chips were printed as a unibody device with two internal LAMP reactors supporting two simultaneous LAMP amplification reactions (Figure 5A,B). The hard–soft 3D-printed screw sealers (shown in Figure 5A) were designed and fabricated to seal the LAMP amplification reactors avoid the use of adhesives and tape sealing. Each amplification reactor has one inlet and outlet port which were designed inside one threaded screw endcap (Figure 5A,B). This allows sealing of two ports with single hard–soft 3D-printed threaded screw (Figure 5A,B). The dimensions of the printed chip (Figure 5B) are (L × W × H in cm) 2.8 × 2.0 × 0.3, and for threaded screw end cap 1 cm in diameter, 0.8 cm in height. Internal amplification reactors are 0.8 mm in height with 24 µL of reagent volume capacity. The inlet and outlet ports are 1.2 mm in diameter that allows injection of LAMP amplification buffer using a 100–200 µL micropipette. 

**Figure 6:**
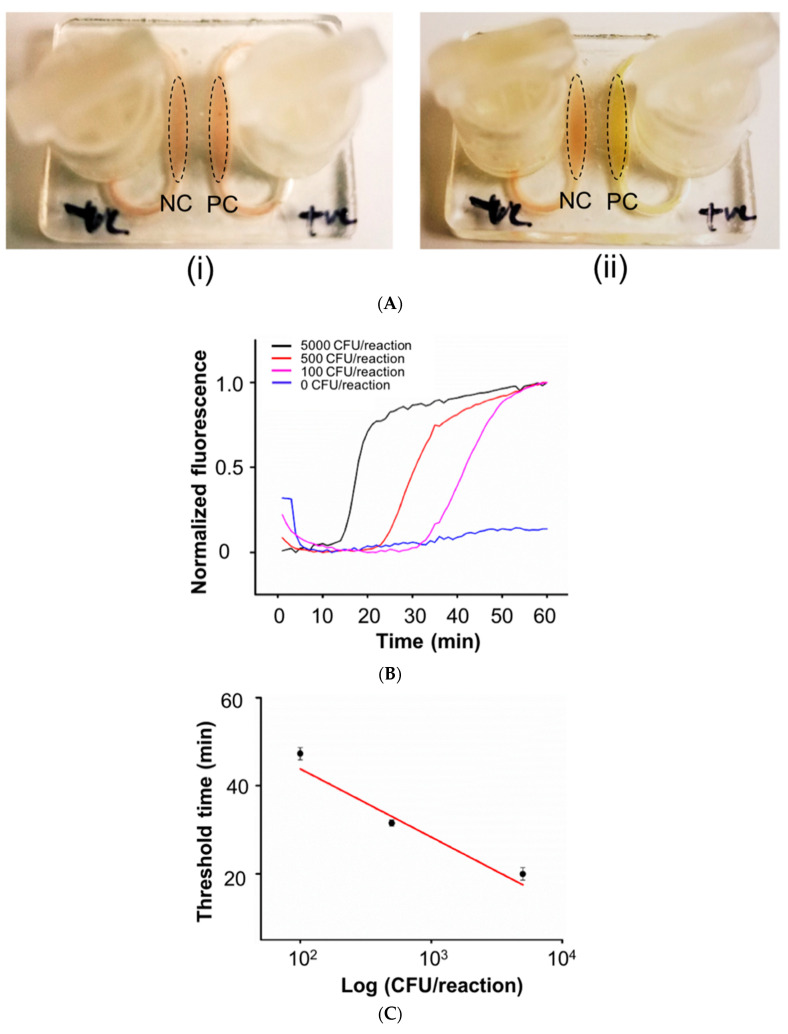
(**A**) Colorimetric LAMP assay of *S. Thyphi* bacterial DNA in our hard–soft hybrid microfluidic chip (i) before and (ii) after LAMP amplification. (**B**) Real-time fluorescence monitoring of LAMP amplification of S.Thyphi bacterial DNA with 0, 100, 500, 5,000 CFU per reaction. (**C**) Threshold time Tt (in minutes), as a function of function of log S.Thyphi bacterial DNA concentration (expressed in terms of CFU per reaction) (n = 3).

Due to high optical clarity and low autofluorescence of the SLA-3D-printed chip, we can use either colorimetric or fluorescence-based detection for our on-chip LAMP assay, as described in the methods section. As shown in Figure 6A, a positive test result was indicated by color change from pinkish to yellow color in presence of positive sample, whereas a negative test result did not show any similar color change after LAMP incubation. To monitor the fluorescence signals of LAMP amplification in real-time, intercalating dye (e.g., Eva^®^Green) was added the LAMP reaction solution. During LAMP reaction incubation, fluorescence images of the microfluidic reactors were recorded in real-time by a DinoCapture™ USB microscope. Image analysis with a MATLAB™ program integrates fluorescence intensities for normalized fluorescence intensity vs time curves for each reactor (Figure 6B). Quantification of the amplification process for three concentrations serves as a calibration curve, where threshold time (*Tt*) is the time to reach 50% of saturation value of the amplification process. Figure 6C shows the linear calibration plot of the logarithm of the amount of spiked gDNA (in terms of CFU per reaction) *vs Tt* in minutes, from which the amount of gDNA target in the sample can be inferred by the threshold time. With our hard–soft microfluidic LAMP chip, we detected *S. Thyphi* bacterial DNA with a sensitivity 100 CFU/reaction. No false positives were observed in negative controls and good RSD’s (Relative Standard Deviations) were found ranging from 2 to 8% (n = 3). In our experiment, no liquid leakage was observed in the hard–soft hybrid microfluidic chip. 

## 4. Conclusions

This work demonstrates the extension of widely available 3D-printing methods for prototyping microfluidic components and devices with increased functionality whereby a hard material and a soft material can be integrated into 3D-printed components and devices. The sequential combination of SLA printing of hard materials and FDM printing of soft materials enables ‘hybrid’ hard/soft material devices with sealing features, flexible diaphragms for fluid actuation, quick-connect fluid coupling, and leak-tight, convenient inlet ports for sample introduction/sealing. Thus, miniature fluidic devices, and especially in vitro medical diagnostics devices, can be quickly prototyped, validated and customized for a wide range of applications including immunoassays and nucleic acid amplification tests. In the later application, the printed devices are shown to be compatible with enzymatic amplification and optical detection (colorimetry or real-time fluorescence) as needed for such tests on the basis of thermal stability, chemical inertness and biocompatibility (minimal surface adsorption of reagents), optical transparency and low autofluorescence, and leak-free, twist-type, screw-sealing, which can be heated (65 °C) for 1-h operation. With our microfluidic LAMP chip, we detected *S. Thyphi* bacterial DNA with a sensitivity 100 CFU/reaction. The extension of the hybrid prototyping method described here to more than two materials and more sophisticated structures would not appear to present any insurmountable difficulties.
